# Unveiling Monkeypox: A Rare Case of Sexual Transmission in Saudi Arabia

**DOI:** 10.7759/cureus.47785

**Published:** 2023-10-27

**Authors:** Ali Alsaeed, Abdullah AlKhalaf, Fatimah Al Matar, Saleh AlRamadan, Ali Al Muhaif, Zahra Marzooq, Zainab Alaali

**Affiliations:** 1 Infectious Disease Division, Department of Internal Medicine, Dammam Medical Complex, Dammam, SAU; 2 Department of Internal Medicine, Dammam Medical Complex, Dammam, SAU; 3 Internal Medicine Residency Program, Department of Internal Medicine, Dammam Medical Complex, Dammam, SAU

**Keywords:** hiv testing, hiv, viral infection, std, monkeypox

## Abstract

This case report describes the presentation, management, and clinical course of monkeypox in a 30-year-old female with multiple sexual partners admitted to Dammam Medical Complex, a major hospital in eastern Saudi Arabia. The patient presented with a rash on her inner thighs and vagina, accompanied by subjective fever and itching. A biopsy confirmed the diagnosis of monkeypox. Despite the absence of complications, the patient was isolated and received conservative therapy under the care of the infectious disease team. This case report highlights the effectiveness of conservative therapy in managing monkeypox, testing another possibility of sexually transmitted disease, and emphasizes the role of the infectious disease team in providing comprehensive care to patients with infectious diseases.

## Introduction

Monkeypox is a rare viral infection caused by the monkeypox virus. It is a double-stranded DNA virus belonging to the Orthopoxvirus family. It is primarily found in Central and West African countries. Although it used to be rare, in the past few years, multiple countries have reported cases of the disease outside of Africa. This is attributed to international travel, zoonotic transmission, and the potential for transmission via sexual routes [1]. 

Although the virus mainly spreads from animal to human, its human-to-human spread is also possible. It is more likely to spread by coming in close contact with someone infected, like when having sex [2]. Here, we present a case of monkeypox in a female with multiple sexual partners, highlighting the importance of timely diagnosis, isolation, and management by the infectious disease team.

Saudi Arabia had already registered confirmed cases by August 2022 [3,4], and Dammam Medical Complex has reported more than eight cases. This case was the first to be diagnosed at our center in August 2023.

## Case presentation

Ms. X, a 30-year-old female with multiple sexual partners, presented to Dammam Medical Complex with a one-day history of a rash on her inner thighs and vagina. She reported subjective fever and itching before the rash appearance.

The patient reported skin lesions that started in the genital area one day before her admission, the lesions appeared on her vagina and inner thighs, and she also experienced a low-grade fever.

**Figure 1:**
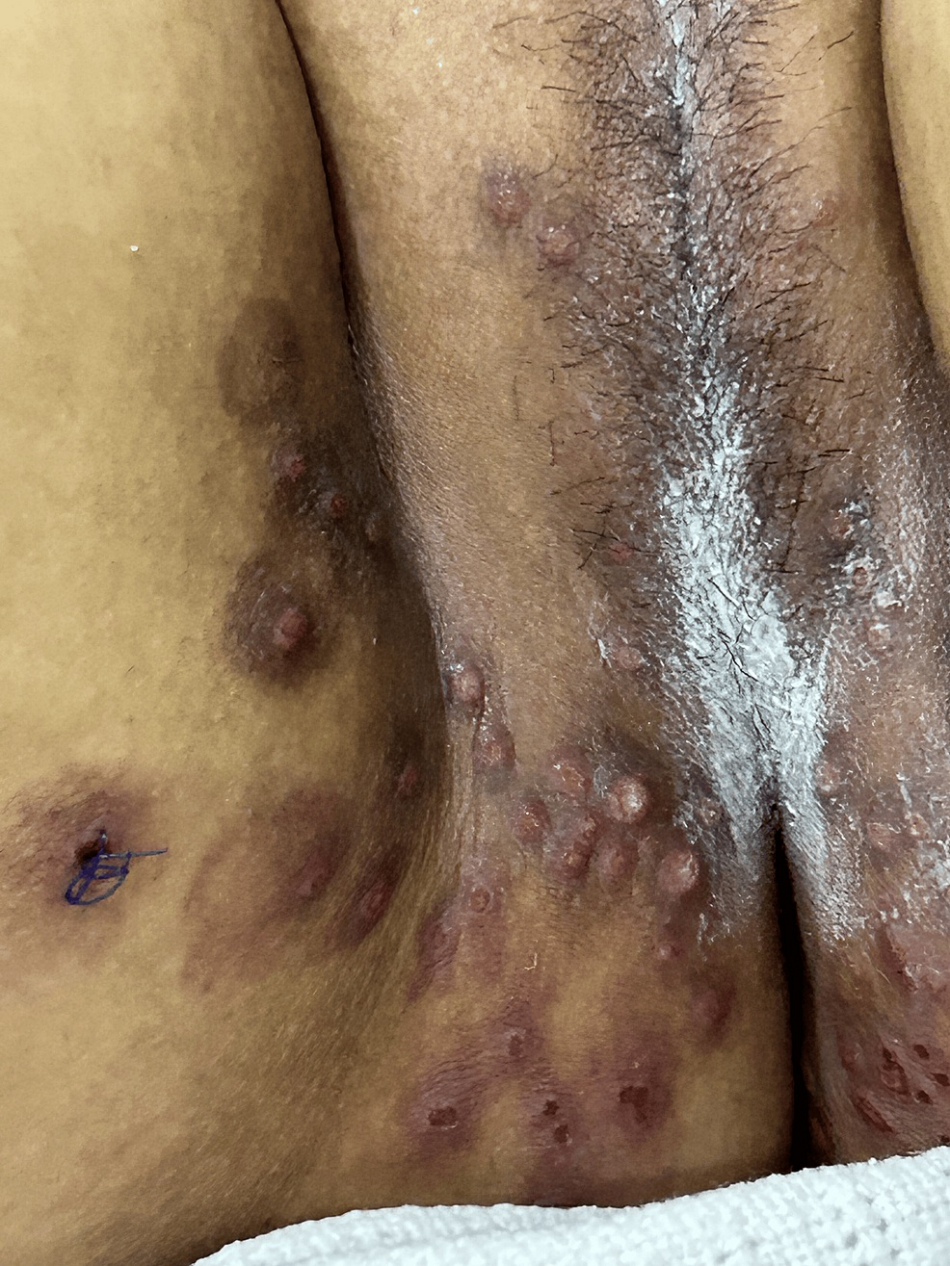
Admission rash after the biopsy was taken.

**Figure 2:**
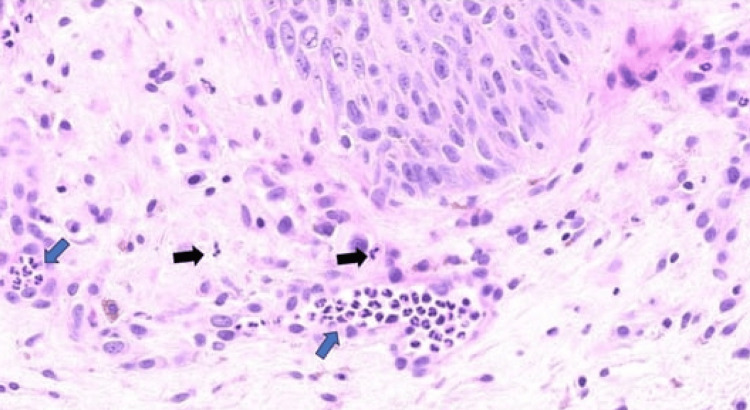
During the pustular stage of monkeypox, there is a presence of dermal neutrophilic infiltration, which is indicated by black arrows. Additionally, these neutrophils can also be seen within the capillary vessels, as denoted by the blue arrow.

On examination, multiple umbilicated pox-like facial and genital lesions at the same stage but more vesicular were observed, predominantly localized to the inner thighs and involving the vaginal mucosa, with palpable lymphadenopathy in the inguinal area (Figures 1-2).

The patient provided four skin samples, which were then sent to the Saudi Ministry of Health National Laboratory for an monkeypox polymerase chain reaction (PCR) test. Within 24 hours, the PCR result returned positive. 

During admission, the patient was found to have a negative HIV test. However, she tested positive for syphilis, suggesting sexual route transmission of monkeypox along with other sexually transmitted diseases (STDs).

Management and clinical course

Upon diagnosis, the patient was immediately isolated in an appropriate isolation room to prevent the spread of the infection. The infectious disease team provided comprehensive care, focusing on conservative therapy. The patient was monitored closely for any signs of complications, such as meningoencephalitis or pneumonia, which fortunately did not develop throughout her 10-day stay.

Conservative therapy included meticulous wound care and hygiene practices. The patient was advised to maintain strict hand hygiene and avoid scratching or manipulating the lesions to prevent secondary infection. Emollient creams were applied to alleviate itching and provide symptomatic relief. Systemic antiviral therapy was not initiated as the patient remained vitally stable and did not exhibit signs of severe disease [5].

The patient did receive ceftriaxone and azithromycin for her STDs.

**Figure 3:**
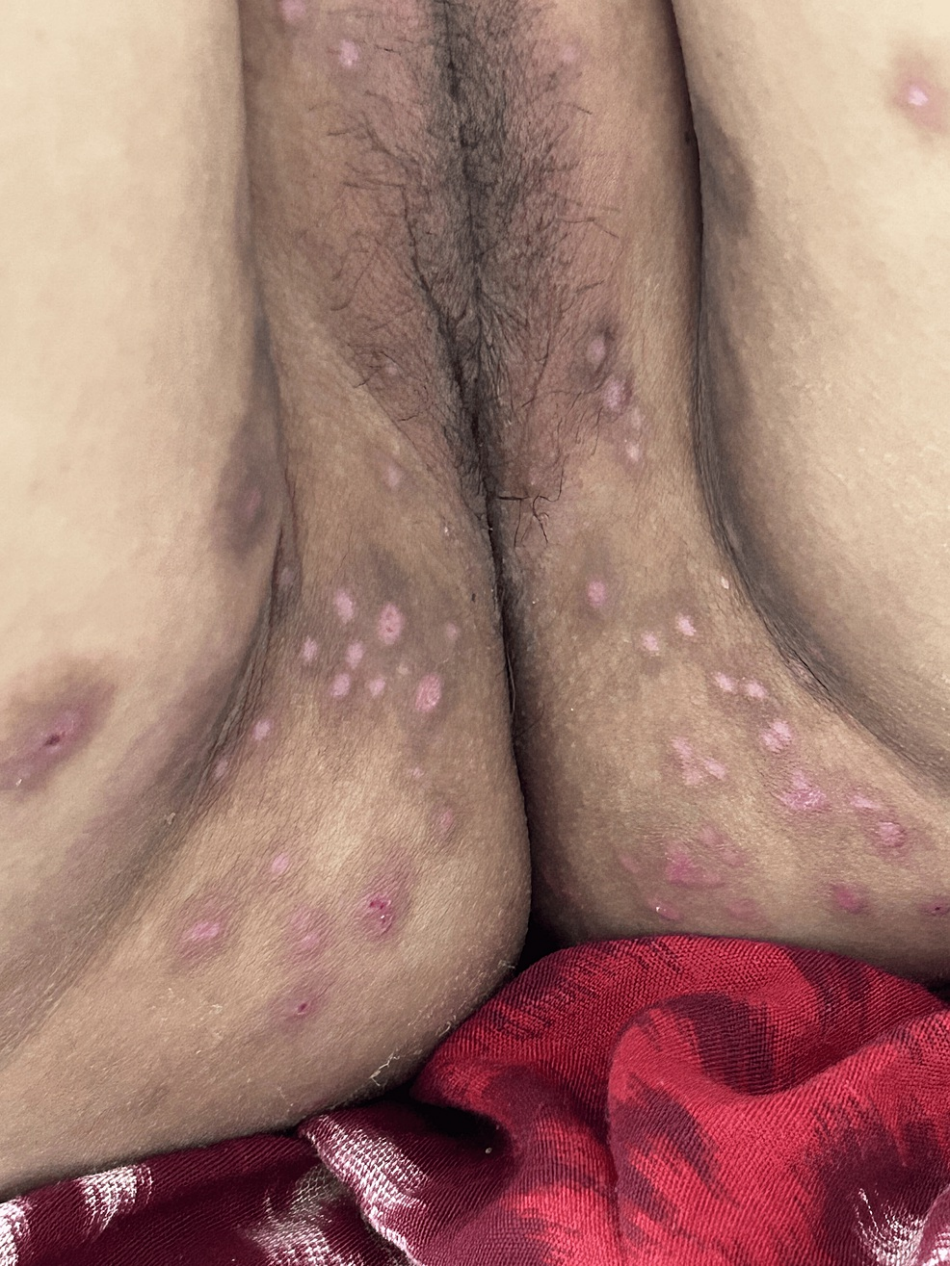
Monkeypox skin lesions scraped, indicating a healing process.

During the hospitalization, the patient's symptoms gradually improved. The rash began to fade, and the itching subsided (Figure 3). Vital signs remained stable throughout treatment. Regular monitoring of the patient's condition, including daily assessments of the rash and vital signs, was conducted by the infectious disease team.

## Discussion

This case report demonstrates the successful management of a female with multiple sexual partners diagnosed with monkeypox through conservative therapy. The absence of complications, such as meningoencephalitis or pneumonia, highlights the importance of early diagnosis and prompt initiation of appropriate isolation measures, early detection, and possible co-infection with sexually transmitted disease. The patient's adherence to meticulous wound care and hygiene practices likely contributed to her favorable clinical course.

The effectiveness of the infectious disease team's care is evident in the patient's stable condition throughout the hospitalization period. The team's expertise in managing infectious diseases, adherence to infection control protocols, and close monitoring of the patient's clinical progress played a crucial role in achieving positive outcomes.

It is important to note that there is a need for prospective studies of antivirals for monkeypox. The management of monkeypox poses unique challenges, even for well-resourced healthcare systems.

Prolonged viral shedding after skin lesion resolution challenges current infection prevention and control guidance [6,7].

## Conclusions

This case report underscores the significance of early diagnosis, isolation, and comprehensive care provided by the infectious disease team in managing cases of monkeypox. Conservative therapy, including meticulous wound care and hygiene practices, can effectively treat uncomplicated cases. Further studies are warranted to explore the optimal management strategies for monkeypox and assess the long-term outcomes of patients with this infection.
